# Clerical Habits

**Published:** 1858-07

**Authors:** 


### CLERICAL HABITS.

An educated and useful clergyman, after swallowing a good-sized apothecary's shop, writes us, that instead of getting better, he is becoming more of an invalid. The wonder is, that he is not dead long ago. We record part of his letter for the benefit of our clerical subscribers.

"I am in doors from half past eight to two P. M., and am engaged in study from dark until twelve o'clock at night. I drink very largely of cold water or milk at my meals; I am in the habit of eating between breakfast and dinner, and also before I go to bed at night. In 1847, I had my palate cut off; I next had the nitrate of silver applied; then used chlorate of potash; I next gargled my throat with sulphate of copper; was induced to go to the Alum Springs in Virginia, and have been taking senna, sulphur, and blue pill, every other night, and have been troubled with costiveness and piles."

As none of these things cured him, he applied to us. If this gentleman had ordered his habits of life aright, he would have been well long ago; but not doing that, he has been a sufferer for twelve long years. But here is an intelligent man stuffing himself with food and physic for twelve years, apparently not reflecting that nothing would cure a man who ate three times a day regularly, and between meals, with a lunch at midnight. And this is the history of many clergymen whose usefulness is curtailed, if not cut short off, and their whole existence embittered by every variety of ailment, merely for the want of a little knowledge, such as this Journal imparts, from time to time. The man who would circulate a thousand copies of our Journal for a year among the clergy of this land, would do a greater good for humanity and the Church, than by erecting the costliest monuments to dead heroes.

---

Sleeping-rooms should never be papered, and, most of all, with paper having any green color, whatever paper-makers may say to the contrary.

